# Epstein-Barr Virus Infection in Gastric Remnant Carcinoma and Recurrent Gastric Carcinoma in Qingdao of Northern China

**DOI:** 10.1371/journal.pone.0148342

**Published:** 2016-02-09

**Authors:** Shuzhen Liu, Zhenzhen Zhao, Lu Han, Song Liu, Bing Luo

**Affiliations:** 1 Department of Blood Transfusion, the Affiliated Hospital of Qingdao University, 16 Jiangsu Road, Qingdao, 266003, China; 2 Department of Medical Microbiology, Qingdao University Medical College, 38 Dengzhou Road, Qingdao, 266021, China; 3 Department of Clinical Laboratory, the Affiliated Hospital of Qingdao University,16 Jiangsu Road, Qingdao, 266003, China; University of Nebraska—Lincoln, UNITED STATES

## Abstract

**Background:**

Epstein-Barr virus (EBV) is associated with a subset of gastric carcinoma which was defined as EBV associated gastric carcinoma (EBVaGC). The proportion of EBVaGC in gastric remnant carcinoma (GRC) which occurs in the intact stomach five or more years after gastric surgery for benign disease is significantly higher than that in conventional gastric carcinoma (CGC). The infection of EBV in recurrent gastric carcinoma (RGC) with local anastomotic recurrence is poorly understood.

**Methods:**

53 cases of GRC and 58 cases of RGC were analyzed for the presence of EBV, and the variants of EBV Encoded RNAs (EBER), EBV Nuclear Antigen 1 (EBNA1) and Latent Membrane Protein 1 (LMP1) gene in both groups were investigated.

**Results:**

Thirteen (24.5%) out of 53 GRC cases and 3 (5.2%) out of 58 RGC cases were identified as EBVaGCs. In 17 paired RGC cases, only one case was classified as EBVaGC in both times specimen. Another one case was identified as EBVaGC in the primary gastroectomy specimen while the recurrent gastric cancer was not. The third EBVaGC in RGC was identified while the primary gastric cancer was not EBVaGC. In GRC and RGC cases, type 1, type F, EB-6m, V-val subtype, del-LMP1 were predominant type or variants, accounting for 10(76.9%) and 2(66.7%), 13(100%) and 3(100%), 13(100%) and 3(100%), 9(69.2%) and 3(100%), 12(92.3%) and 3(100%), respectively. However, Type C was the predominant type in GRC accounting for 9(69.2%) cases while type D was the predominant one accounting for 2(66.7%) cases in RGC.

**Conclusions:**

The prevalence of EBVaGc in GRC and RGC was significantly different. The distributions of these variants were similar to each other in the two groups which indicated that there were no more aggressive EBV variants in EBVaGC in GRC compared with that in RGC.

## Introduction

Epstein-Barr virus is a tumorigenic herpes virus that infects over 90% of the adults worldwide. Although the most individuals carry the virus as a lifelong asymptomatic infection, EBV has potent transforming ability and is associated with human malignancies such as Burkitt’s lymphoma (BL), Hodgkin lymphoma (HD) and nasopharyngeal carcinoma (NPC) [1–3]. In the past several years, EBV involvement has been demonstrated in gastric carcinoma (GC) with prevalence varying from 1.3% to 20.1% in different countries [4–6]. The previous study in our laboratory showed that the prevalence of EBVaGC in Northern China where is a non-endemic area of NPC was 7.0%(13/185)[7]. Larger investigation demonstrated the similar rate(6.1%, 102/1678) of EBVaGC in GC in Northern China[8].

The exact role of EBV in the pathogenesis of EBV-associated malignancies remains to be determined. The fact that EBV infection is ubiquitous in the world but the incidence distribution of EBV-associated malignancies differs in geographic regions raises the possibility that particular EBV strains contribute to the development of specific EBV-associated malignancies[9–13]. The latent infection of EBV in EBVaGC belongs to type I or II, in which EBERs, EBNA-1, BARTs and BART miRNAs are expressed and approximately half of EBVaGC cases express LMP-2A [14, 15].

According to sequence divergence within the EBV nuclear antigens (EBNA-2, -3A, -3B, -3C) and the different capacity to transform B lymphocytes into a state of continuous immortalization, EBV can be classified as genotypes A or B, also known as type 1 or 2[16]. Compared with type A, type B has a lower transforming efficiency, a poorer initial outgrowth, and higher cell density dependence for cell viability in vitro [17]. It has been reported that the incidence of EBV infection varies geographically [18]. Type A is predominant in Southern China, Tunisian, Slovenia, Japan, and North America, whereas type B has been found mainly in Alaska[19–23]. A further attempt to identify polymorphisms of virus isolates in the *Bam* HI I and *Bam* HI F region of the EBV genome resulted in the so-called type C/D and type F/f, which represent single nucleotide polymorphisms at *Bam* HI sites on the viral genome. Types C and F do not have a *Bam* HI restriction site in the *Bam* HI W1/I1 region and *Bam* HI F region, respectively, while types D and f possess this site in the corresponding region. Type C prevails in NPC patients from Southern China-an endemic area of NPC, while type D is prevalent in the United States [20, 24, 25]. The f variant appears to be more frequent in NPC patients from Southern China than in healthy Chinese individuals, suggesting that this variant may be tumor associated[26, 27]. But a previous study by Cui in our laboratory showed that the frequency of type f strains in NPC was significantly lower in Northern China than in Southern China, suggesting that EBV strains derived from the NPC patients may reflect geographic distribution rather than being NPC restricted [28]. As for the polymorphisms of EBNA1 in Northern China, three major patterns of the EBNA1 variations, V-val, P-thrV and V-leuV, were observed, and V-val was the most common subtype in all the three groups, followed by P-thrV and V-leuV[29]. Analysis of LMP1 gene exon3 leads to two variants, wt-LMP1 and del-LMP1. Del-LMP1 variant, which has a 30-bp sequence deletion when compared with the wt-LMP1 variant, predominates in Chinese and Taiwanese population[30]. There are three main distinct variants of EBER genes, designated as EB-6m, EB-8m, and EB-10m. EB-6m is the predominant variant in Northern China[8].

Gastric remnant carcinoma (GRC) is defined as a carcinoma occurring in the gastric stump at least 5 years after surgery for benign diseases such as gastric ulcer and duodenal ulcer[31, 32]. It has been reported that the proportion of EBVaGC in GRC was apparently higher than that in conventional gastric carcinoma (CGC) in Japan (27.1% *vs* 6.4%)[33], Netherlands (35% *vs* 8%)[34], Korea (29% *vs* 6%)[35] and Southern China (30.8% *vs* 6.7%)[36]. The infection of EBV in recurrent gastric carcinoma (RGC) was seldom being researched as an independent group. RGC is cancer that has recurred after it has been treated. The cancer may come back in the stomach or in other parts of the body such as the liver or lymph nodes. Chang has investigated the association between the infection of EBV and the metachronous GRC, the prevalence is similar with that in CGC (8% *vs* 6%)[35]. To our best knowledge, there was no report on the prevalence of EBV infection in RGC.

The aim of the present study is to investigate the prevalence of EBV in GRC and RGC in Qingdao of Northern China. EBV genotype and latent gene variants of EBER, EBNA1 and LMP1 were also analyzed in EBVaGC in both groups.

## Materials and Methods

### Specimens and DNA extraction

This study was approved by the Medical Ethics Committee of the Medical College of Qingdao University. All patients involved in the present study gave oral or written informed consents for the use of tissue samples for research. The oral informed consents were obtained from little number patients through telephone due to the long time after their discharged. The consents were documented through telephone recording. The Medical Ethics Committee also approved this consent procedure. Fifty-three GRC and Fifty-eight RGC paraffin-embedded tissues were collected from the Affiliated Hospital of Qingdao University and Qingdao Municipal Hospital from January 1, 1996 to June 30, 2015. Seventeen RGC cases with primary and secondary specimens were examined for EBV infection. Histology of the GRCs and RGCs was classified by the predominant histological pattern as intestinal- and diffuse- type according to the Lauren classification[37]. The infection of EBV in GC was determined by EBER1 in situ hybridization, as described previously [14]. The cases with EBER1 positive signals in carcinoma cells were classified as EBVaGCs. QIAamp DNA FFPE Tissue kit (QIA-GEN GmbH, Hilden, Germany) was used to extract the DNA from paraffin-embedded tumor tissues.

### PCR and sequencing of EBER and EBNA1 genes

The EBV EBER and EBNA1 genes were amplified by the nested PCR technique. All of the specific oligonucleotide primers and the sizes of the PCR products in this study are listed in Table 1. In each set of PCR reactions, DNA from EBV-positive B95-8 cell lines was used as a positive control, and nuclease-free distilled water served as a negative control. For amplification, the first-round PCR was performed in a total volume of 25 μl containing 1×PCR reaction buffer, 100 ng of genomic DNA, 0.5 μM each primer, 200 μM each deoxyribonucleotidetriphosphate, and 1 U Pfu Taq polymerase (TaKaRa Biotechnology Co. Ltd., Kyoto, Japan). PCR amplification was performed with an initial denaturation at 94°C for 5 min; 35 cycles of denaturation at 94°C for 30 s, annealing at 55°C for 30 s, extension at 72°C for 1 min; and a final elongation step at 72°C for 10 min. EBER-OS1 combined with EBER-O1 and EBNA1-1 combined with EBNA1-2 was used as the outer primers for EBER and EBNA1, respectively. When necessary, 2 μl of the PCR product were taken for a second round of PCR, using the internal primers EBER-NS1 combined with EBER-N1 and EBNA1-3 combined with EBNA1-4. In order to prevent contamination, several measures were taken, such as frequently changing gloves and cleaning the equipment, using aerosol-resistant pipette tips for PCR, and performing different procedures in separate areas. The PCR products were analyzed using an ABI 3730 DNA sequencer to confirm variants identity.

**Table 1:** Primers used in EBER, EBNA1 and LMP1 amplification and EBV genotyping.

Name of Primers	Sequence(5’-3’)	Size of PCR products	B95-8 coordinates
**1/2 types**			
Type-F1	AGGGATGCCTGGACACAAG		
Type-R1	GTGCTGGTGCTGCTGGTGG		
Type1-1	TCTTGATAGGGATCCGCTAGGATA	Type 1 = 497bp	—
Type1-2	ACCGTGGTTCTGGACTATCTGGAT		
Type2-1	CATGGTAGCCTTAGGACATA	Type B = 150bp	—
Type2-2	AGACTTAGTTGATGCCCTAG		
F/f types			
Type F-1	TCCCACCTGTTACCACATTC	Type F = 198p	—
Type F-2	GGCAATGGGACGTCTTGTAA	Type f = 127+71bp	
C/D types			
Type C-1	ACCTGCTACTCTTCGGAAAC	Type C = 206bp	—
Type C-2	TCTGTCACAACCTCACTGTC	Type D = 130+76bp	
EBER-OS1	AATGAGGGTTAGCATAGGC	718bp	6513–6531
EBER-O1	GTCACAGAATTGATTGGCA		7230–7212
EBER-NS1	GTCTGTCTTGAGGAGATGT	600bp	6585–6603
EBER-N1	TTTGTGTTGTAGGGGTAGC		7184–7166
EBNA1-1	TAGTCAGTCATCATCATCCG	843bp	109104–109123
EBNA1-2	GGGATTTATTCTTTAGTGCG		109946–109927
EBNA1-3	GCCATTTTTCCACCCTGTAG	745bp	109158–109177
EBNA1-4	ATTGAGGGCGTCTCCTAACA		109902–109883
Del-LMP1	TTGAAAACAAAGGAGGTGAC	Wt-219bp	168341–168322
	AGCCTATGACATGGTAATGC	Del-189bp	168123–168142

### PCR for LMP1 deletion

The C-terminal 30 bp gene deletion of LMP1 was tested by the PCR technique, as previously reported [38]. The primer sequences and the size of PCR products were also shown in Table 1. The condition for DNA amplification and detection of the PCR products were the same as EBV genotypes.

### Definition of EBV genotype

**Fig 1:**
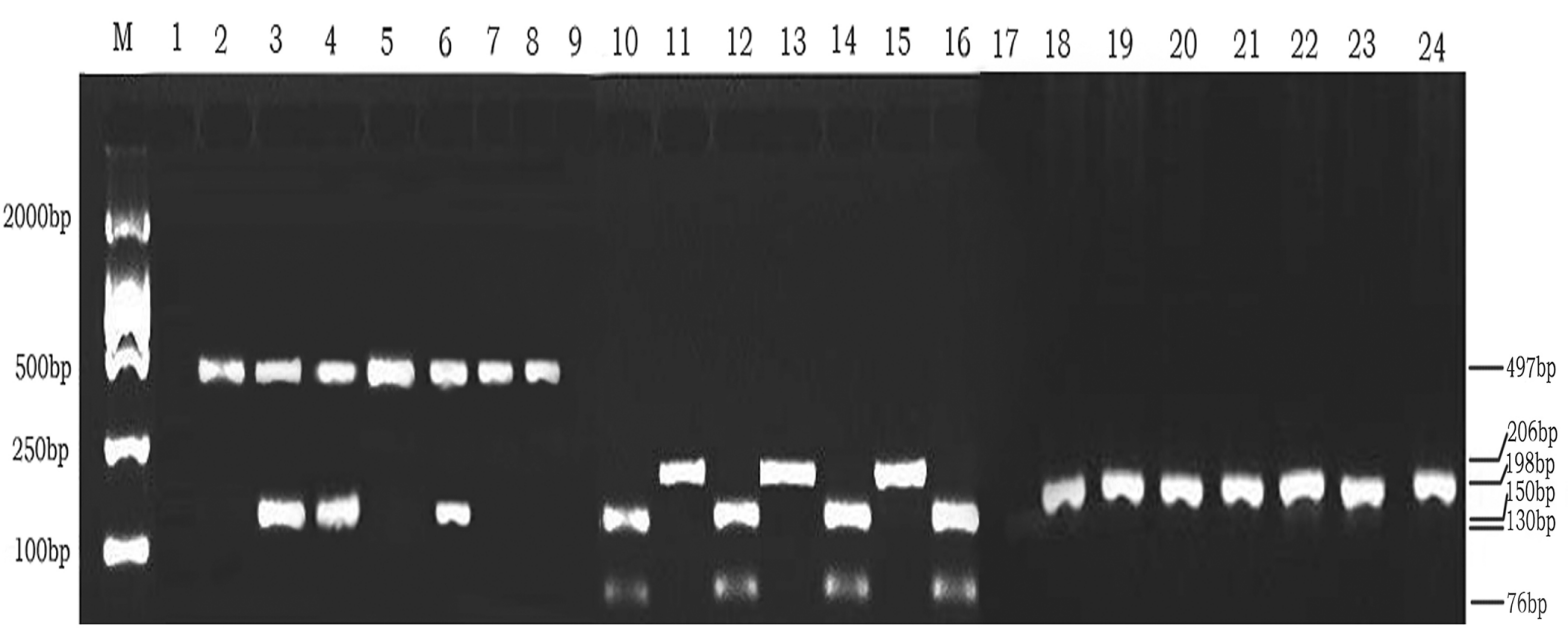
PCR analysis for EBV genotyping for 1/2,F/f,C/D. **Lanes 1–8:** Lane M, DL 2000 DNA Marker; lane 1, negative control; lane 2, B95-8 prototype (Type 1); lane 5,7,8, representative cases of Type 1; lane 3,4,6,representative case of co-infection with Type 1 and 2; no subtype 2 was detected in the study. **Lanes 9–16:RFLP analysis with BamHI restriction enzyme digestion after PCR amplification at BamHI W1/I1 region.** Lane M, DL2000 DNA Marker; lane 9, negative control; lane 10, Raji prototype (Type D); lane 11, 13 and 15, representative cases of Type C strains; lane 12,14,16, representative cases of Type D strains. **Lanes 17–24: RFLP analysis with BamHI restriction enzyme digestion after PCR amplification at BamHI F region.** Lane M, DL2000 DNA Marker; lane 17, negative control; lane 18, B95-8 prototype (Type F); lane 19–24 representative cases of the Type F strains; No type f strain was found in the present study.

The identification of the EBV genotypes 1 and 2 was carried out by determining the 3’ sequence divergence of EBNA2 gene by the nested PCR method as described previously in Ref [39]. The primers Type-F1 and Type-R1 were used as the outer primers (Table 1). In the second round of amplification, 497bp for genotype 1 with the primers Type1-1 and Type1-2, and 150bp for the EBV genotype 2 with the primers Type2-1 and Type2-2 (Table 1 and Fig 1).

EBV Bam HI F region and Bam HI W1/I1 region were carried out by PCR with restriction fragment length polymorphism(RFLP). The primer sequences and the size of PCR products are shown in Table 1. PCR products of the Type C/D and F/f were digested with the Bam HI restriction enzyme reactions of a 20-μl reaction mixture (containing 10μl of PCR products, 1 × reaction buffer and 10 units of Bam HI) at 30°C for 4 h, the DNA products were analyzed on 2% agarose gel, and then visualized by ethidium bromide staining.

### Statistical analysis

Chi-square test or the Fisher’s exact test was performed to determine the differences in distribution of EBV genotype and variations in EBVaGC both in GRC and RGC. Differences were considered significant when P < 0.05. SPSS 18.0 statistical software (SPSS, Chicago, IL) was used for statistical elaboration.

## Results

### Determination of EBV positive specimens

In the present study, 53 GRC and 58 RGC were investigated for the presence of EBER-1. 13(24.5%) and 3(5.2%) cases were identified as EBVaGC in GRC and RGC group, respectively. There were 46(86.8%) male cases and 7(13.2%) female cases in GRC group, respectively. Ten male cases (76.9%) and 3 female cases (23.1%) were identified as EBVaGCs. The mean age was 63.9±11.0 years. There were 44 male cases (75.9%) and 14 female cases (24.1%) in RGC group. The mean age was 56.2±11.6 years. All the three EBVaGCs were male cases. In GRC group, 44 cases and 9 cases were identified as diffuse- and intestinal type, respectively. Among the thirteen EBVaGCs in GRCs, 11 and 2 cases were classified as diffuse- and intestinal- type, respectively. In RGC group, 47 cases and 11 cases were identified as diffuse- and intestinal type, respectively. Two EBVaGCs were classified as diffuse- type and the other one was identified as intestinal type. The difference is not significant in both groups about the clinicopathologic characteristics. There are significant differences between GRC and RGC group in age and lymph node metastasis. The clinicopathologic characteristics of EBVaGC and EBVnGC (EBV negative gastric carcinoma) in GRC and RGC are listed in Table 2.

**Table 2:** Clinicopathologic characteristics of EBVaGC and EBVnGC in GRC and RGC.

Variables			GRC				RGC		
	Total^a^	EBVaGC	EBVnGC	P^a^	Total^b^	EBVaGC	EBVnGC	P^b^	P^c^
**Gender**				0.23				1.00	0.14
**Male**	46	10	36		44	3	41		
**Female**	7	3	4		14	0	14		
**Age(years)**				0.09				1.00	0.00^※^
**≤60**	17	7	10		40	2	38		
**>60**	36	6	30		18	1	17		
**Smoking History**				0.56				1.00	0.79
**Yes**	36	8	26		38	2	36		
**No**	17	5	11		20	1	19		
**Reconstruction Style**				0.004^*^				0.60	0.55
**B I**	31	3	28		30	1	29		
**B II**	22	10	12		28	2	26		
**Mean±SD(years)**	63.9±11.0	61.0±8.6	64.0±14.0		56.2±11.6	55.7±5.6	56.1±12.1		
**Histology**^d^				1.00				0.47	0.97
**Diffuse type**	44	11	33		47	2	45		
**Intestinal type**	9	2	7		11	1	10		
**Lymph node metastasis**				1.00				1.00	0.00^※^
**Yes**	40	10	30		19	1	18		
**No**	13	3	10		39	2	37		

a: EBVaGC *Vs* EBVnGC in GRC

b:EBVaGC *Vs* EBVnGC in RGC

c: GRC *Vs* RGC

d: Lauren classification EBVnGC: EBV negative GC

### The distribution of genotypes and variants in EBVaGC in GRC

Amplification of the six regions of EBV gene was performed successfully in all 16 EBVaGC cases except for EBNA1. EBNA1 was not successfully amplified in four EBVaGC cases. The genotypes of EBV were determined by PCR-RFLP. The type 1, type F, type C, EB-6m, V-val and del-LMP1 variants were predominant among EBVaGC patients, accounting for 10 (76.9%), 13(100%),9(69.2%), 13 (100%), 9 (69.2%) and 12 (92.3%) cases, respectively. The EBV genotypes and variants of latent genes in the EBVaGCs are listed in Table 3.

**Table 3:** Distributions of EBV genotype or variants in EBVaGC of GRC and RGC.

Genotypes/Variants	EBVaGC in GRC		EBVaGC in RGC	
	n = 13	%	n = 3	%
1	10	76.9	2	66.7
2	0	0	0	0
1 and 2	3	23.1	1	33.3
F	13	100	3	100
f	0	0	0	0
F and f	0	0	0	0
C	9	69.2	1	33.3
D	4	30.8	2	66.7
C and D	0	0	0	0
EBER				
EB-6m	13	100	3	100
EB-8m	0	0	0	0
EB-10m	0	0	0	0
EBNA-1				
V-val	9	69.2	3	100
V-thr	0	0	0	0
V-leu	0	0	0	0
NA	4	30.8	0	0
30bp deletion in LMP1				
del-LMP1	12	92.3	3	100
wt-LMP1	1	7.7	0	0
del-LMP1 and wt-LMP1	0	0	0	0

NA: not amplified

### The distribution of genotypes and variants in EBVaGC in RGC

Amplification of the six regions of EBV gene was performed successfully in all 3 EBVaGC cases. Type 1, type F, type D, EB-6m, V-val, del-LMP1 were predominant type or variants in EBVaGC, accounting for 2(66.7%), 3(100%), 2(66.7%), 3(100%), 3(100%) and 3(100%) cases, respectively. The EBV genotypes and variants of latent genes of EBV in EBVaGC in RGC are listed in Table 3.

**Fig 2:**
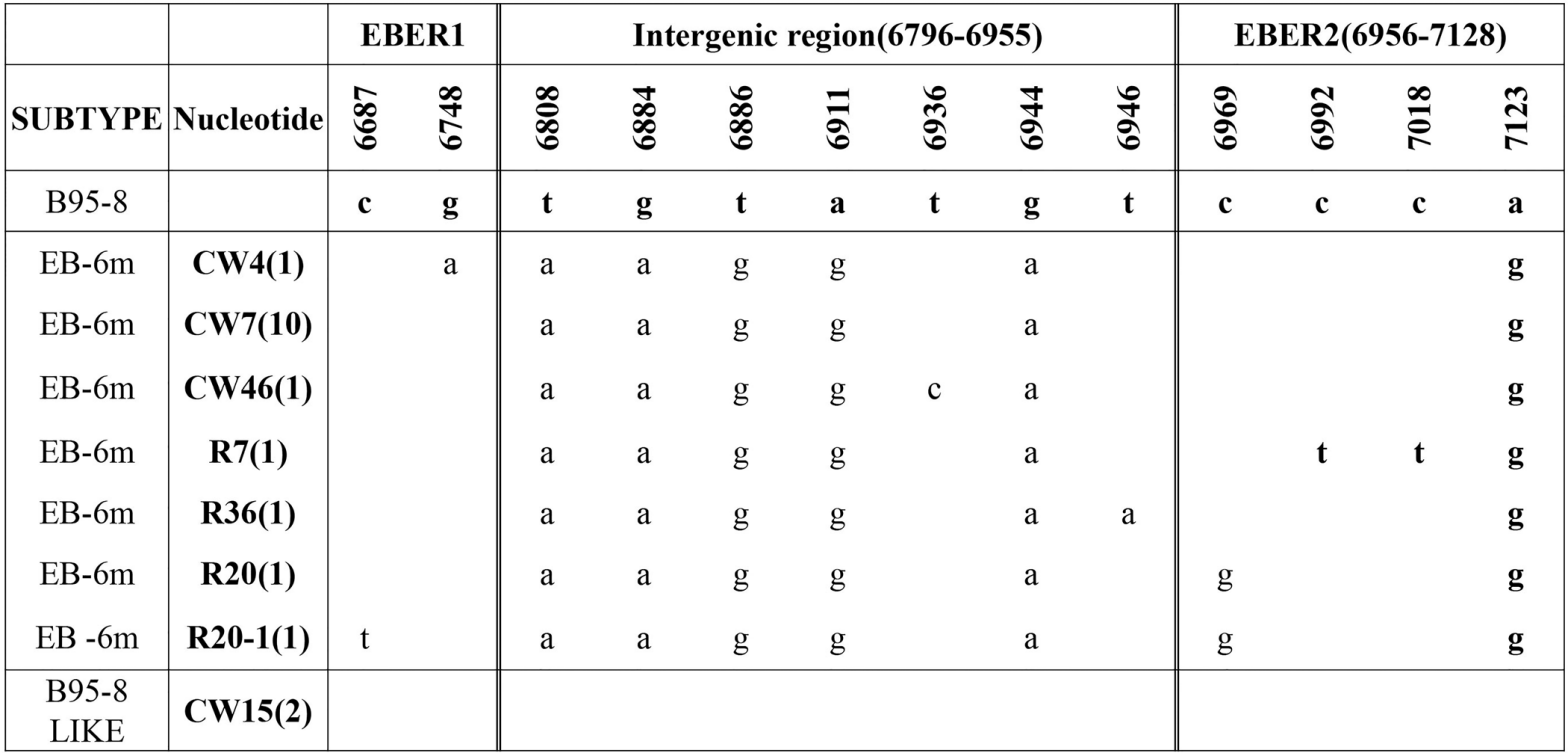
EBER sequence variations in 12 EBVaGC in GRC and 3EBVaGC in RGC. Names in the left column refer to EBER variants identified in this study. Names in the near column refer to the representative isolates, and the numbers in the parentheses after the name denote the number of isolates carried identical sequence with the representative isolate. An asterisk indicates a deletion of a nucleotide. EB-6m was the only variant in the present study. The initial specimen identified as EBVaGC was also being analysed.

**Fig 3:**
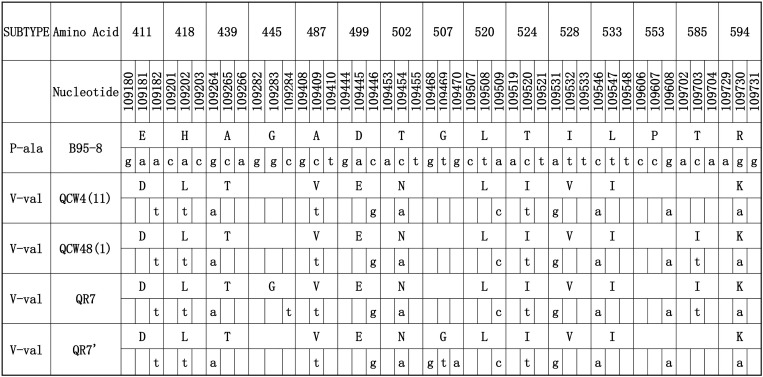
EBNA1 sequence variations in EBVaGC. Numbers across the top correspond to the amino acid positions under which the B95-8 prototype amino acid and nucleotide sequence is listed. Different patterns are noted to the left column, while the specimens showing identical sequences to each other are listed by a representative isolate in the second column. The followed numbers in the parentheses denote the amount of the identical sequences from EBVaGC. Four EBVaGC specimen from GRC were not amplified successfully. V-val was the only variant in all EBVaGC cases. The initial specimen identified as EBVaGC was also being analysed.

**Fig 4:**
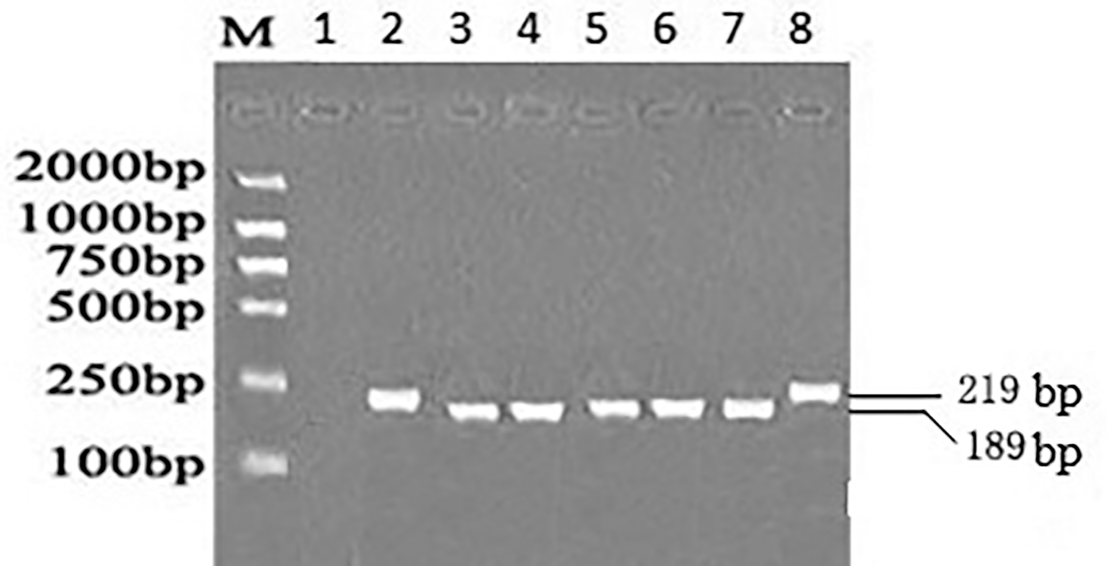
PCR analysis for 30bp deletion in LMP1. Lane M, DL2000 DNA Marker; lane 1, negative control; lane 2 and lane 8, representative cases of wild type of LMP1; lane 3,4,5,6,7 representative cases of del-LMP1.

The detail distributions of EBER and EBNA1 were presented in Figs 2 and 3. EB-6m sequences were found in all 16 EBVaGC cases (Fig 2). These showed five common mutations (positions 6808, 6884, 6866, 6911, and 6944) at 161 bp space region and one mutation (position 7123) at EBER2 genes compared with B95-8. The most common pattern carried 12 consensus sequence changes, including 10 AA changes at residues 411 (Glu→Asp), 418(His→Leu), 439 (Ala→Thr), 487 (Ala→Val), 499 (Asp→Glu), 502 (Thr→Asn), 524 (Thr→Ile), 528 (Ile→Val), 533 (Leu→Ile), and 594 (Arg→Lys) and two silent changes at residues 520 (CTA→CTC) and 553 (CCG→CCA). This consensus sequence (represented by QCW4 in Fig 3) was same to the sequences of published V-val subtype or its subvariants at the sequenced. The PCR products of LMP1 were showed in Fig 4.

### EBV infection in seventeen paired specimens of RGC

In RGC group, seventeen RGC cases with primary operation specimens were investigated. Four specimens from three cases were identified as EBVaGC. In one case both specimens were identified as EBVaGC but there were different EBV genotypes in the two specimens. Another case was classified as EBVaGC only in the specimen of the initial operation. The third case was identified as EBVaGC in the second specimen while the primary specimen was not EBVaGC.

## Discussion

In contrast to BL and NPC, which are endemic in Equatorial Africa and Southeast Asia, respectively, EBVaGC is a non-endemic disease distributed throughout the world[40]. However, there are some regional differences in the incidence of EBVaGC. The incidence of EBVaGC in all cases of gastric cancer is distributed from highest (16–18%) in the USA and Germany to the lowest (4.3%) in China [40, 41]. In the present study, 13 out of 53 cases of GRC and 3 out of 58 cases of RGC were identified as EBVaGC cases. The frequency was 24.5% and 5.2% respectively, and there was significant difference between them. The proportion of EBVaGC in GRC was consistent with previous studies in other areas of the world [33–36]. The prevalence of EBV in RGC was also similar to the proportion of CGC investigated by our laboratory at the same area (7.0%(13/185) and6.1%(102/1678)[7, 8]. This difference in distribution has indicated a strong association between EBV and the remnant stomach.

In the present study, we analyzed EBV polymorphisms in the EBNA2 gene (type 1 or 2), Bam HI W1/I1 region (type C or D), and Bam HI F region (type F or f), and we also sequenced the EBER and EBNA1 of EBVaGC both in GRC and RGC in Northern China. LMP1 genes were analyzed by PCR and electrophoresis.

Based on EBV genotype and RFLP analysis, all EBVaGC samples both in GRC and RGC were detected by PCR. The distribution of EBV genotyping 1/2, C/D and F/f in carcinoma tissues was consistent with those in previous studies except type D in RGC [8, 29, 42]. Types 1, C or F were the most frequent subtypes in GRC while type D was predominant in RGC. Although type D was the predominant subtype in RGC, there was no significant difference due to the very limited case number. These results showed that EBV polymorphisms/variants might have no association with the difference in prevalence of EBV among GRC, RGC and CGC. Other factors may involve the difference among them.

The results in our study showed that V-Val in EBNA1, Em-6 in EBER and del-LMP1 was the predominant variants in EBVaGC both in GRC and RGC. The results were consistent with that both in Southern China and Northern China. One research on variants of EBNA1 in Guangzhou by Chen showed that V-val subtype was the most common variant both in GRC and CGC [43].The del-LMP1 was also the predominant variant in EBVaGC in both GRC and CGC in Guangzhou, Southern China [44]. In Northern China, Wang found that V-val was also the most common subtype not only in NPC but also in EBVaGC and healthy donors[29]. EB-6m of EBER was the predominant variant in 98%(48/50) CGC cases in Northern China[8]. All the information demonstrates that no significant difference in distribution of variants of EBV genes in EBVaGC both in GRC and RGC by comparison with CGC.

Additionally, we also analyzed 17 paired RGC cases which were the same patients with both primary and secondary gastrectomy for gastric carcinoma. Only one case was identified as EBVaGC in samples from primary and secondary gastric cancers. But in the case, the EBV type 1/type 2 and type C/D were different. The specimen for first gastrectomy was classified as mixture type for type 1/2 and type C while the second specimen was identified as type 1 and type D. It may suggest the tumorigenesis caused by different EBV strain. Interestingly, in another case, the primary sample was diagnosed as EBVaGC while the second specimen of the same patient was not EBVaGC. In this distinctive case, it is tempting to suggest that the tumor was dissected completely while the recurrent gastric carcinoma was not associated with EBV. As for the third case, the second specimen was diagnosed as EBVaGC while the initial specimen was not EBVaGC. It may derive from the near lymph node bearing EBV.

In the past years, the injuries of gastric mucosa and/or changes of the microenvironment were considered as the main causes for high proportion of EBVaGC in GRC. Nishikawa found that atrophic change of remnant gastritis in Billroth-II anastomoses is considered to be the carcinogenic background for EBV-positive gastric remnant carcinoma [45]. It has been reported that frequent salty food intake and wood dust and/or iron filings exposure are associated with a higher EBVaGC risk, suggesting that mechanical injuries to the gastric mucosa may be involved in the development of EBVaGC[46, 47]. In the present study, the difference of the reconstruction style between EBVaGC and EBVnGC in GRC was significant that suggested the changes of some physical and chemical factors due to reconstruction style might have facilitated the development of EBVaGC in GRC (P = 0.003). In RGC, the difference was not significant possibly partly due to the limited number of EBVaGC cases (P = 0.55).

It has been reported that the rate of EBV infection in epithelial cells is much higher by co-culturing with EBV-positive B cells than by cell-free infection [48–50]. When most EBV virions bind to primary B cells, they are retained on cell surfaces and transferred to epithelial cells that results in a significant increase of infection compared with cell-free virus infection[51, 52]. All these studies support that efficient EBV transmission into epithelium can be acquired through EBV-infected B cells migrating into the neoplastic stroma or intraepithelial space through cell-to-cell contact[53]. In the present study, the major difference between GRC and RGC is that stomach lymph nodes have not been cleaned for the first surgery. The B cells from residual lymph nodes may give the convenience for EBV involvement in the development of EBVaGC in GRC.

## Conclusions

In the present study, we found that the proportion of EBVaGC in GRC was higher than that in RGC in Northern China. For the first time we studied the paired specimens of RGC. There was no significant difference in EBV types and variants in EBVaGC among the two groups. Other physical and/or chemical factors such as mechanical injuries of mucosa and changes of microenvironment may be involved in the development of EBVaGC. The residual gastric lymph nodes might play a vital role in explaining the higher proportion of EBV in GRC.

Our results showed that no significant difference existed among EBVaGC in GRC and RGC on EBV genotypes and variants. Due to the very limited number of EBVaGC in RGC patients, we cautiously drawed the conclusion. It still remains difficult to find the exact reason to explain the different prevalence of EBV infection in GRC and RGC. Further studies will be needed to elucidate the difference deeply. And great number of patients is also needed to be researched to demonstrate the distributions of EBV genotypes and variants of latent genes.
